# Spinal Caries

**Published:** 1908-01-11

**Authors:** Donald Armour

**Affiliations:** Assistant Surgeon to the West London Hospital and the National Hospital for the Paralysed and Epileptic; Surgeon to the Belgrave Hospital for Children.


### SPINAL CARIES.

By DONALD ARMOUR, B.A., M.B., F.R.C.S., Assistant Surgeon to the West London Hospital and the National Hospital for the Paralysed and Epileptic; Surgeon to the Belgrave Hospital for Children.

A lecture delivered at the National Hospital for the Paralysed and Epileptic.

By spinal caries, which is known also as Pott's disease, I mean tuberculous osteitis affecting any part of the vertebral column. Another name for this condition, which I only wish to mention in order to condemn it, is angular curvature of the spine. It is obvious that this is the name of a symptom only, and one which is a reproach to the previous treatment of the case. For if treatment be carried out properly, and for a long enough period, there should be no angular deformity.

### Pathology.

There is no time to-day to discuss the subject of spinal caries in its entirety; I shall limit myself to a consideration of the disease as we see it here—that is, as giving rise to symptoms connected with the nervous system. There are, however, some points in the pathology of spinal caries which I must mention. The disease differs in no respect from tuberculous disease of the cancellous tissue of bone in other parts of the body. There are the stages of infection, inflammatory reaction, formation of tuberculous nodules, and caseation. In the vast majority of cases the lesion affects the bodies of the vertebræ: rarely it begins in the spinous processes or laminæ. In the atlas and axis the disease may begin in the joints between these bones.

When the seat of infection is the body of a vertebra, one of two places is chosen: either the anterior surface just beneath the layer of compact bone, or else near the epiphyseal plates at the upper or lower surface. Caries has been said to begin in the intervertebral discs: this is difficult to affirm or to deny, for if it ever does so the process rapidly spreads to the bone. Having begun, no matter in what part of the vertebra, the course is exactly the same. A diffuse tuberculous osteitis ensues, going on to caseation and even to pus formation. The disease may be at first limited to one vertebra, or it may start in several vertebræ at the same time. When limited to one, it certainly does not remain so very long, but spreads to others. When more than one are involved the only difference is in the acuteness of the resulting deformity, which has a much more gradual curve if several vertebræ are affected. The natural result of this destruction of bone is the giving way, owing to the superincumbent weight of the head and of the body above the lesion, of the vertebral column. Hence deformity is one of the first results. Another is the narrowing of the spinal canal to a greater or less extent. This is especially true of the dorsal region, where the canal is much smaller than elsewhere. Caries is more frequent in the dorsal or lumbo-dorsal region than in others, and that is where we commonly see the disease in cases brought here. A third result is pressure on the cord itself. I may say at once that you will see cases showing well-marked deformity which have never had any symptoms referable to the spinal cord. That is, increased deformity does not necessarily mean increased pressure on the spinal cord.

On the other hand, many cases with little or no deformity have well-marked nervous symptoms. These symptoms may be due to three main causes: (1) A tuberculous abscess in the body of a vertebra may strip up the posterior common ligament and push it backward so as to encroach on the space of the spinal canal. (2) The presence of bony sequestra may similarly act as a cause of pressure. These two conditions are rarely seen: cases of paraplegia due to bony pressure constitute only 2 per cent. of all cases, but it is necessary to remember the possibility of the occurrence, for the most acute conditions are often due to this cause. The determining cause of pressure by a sequestrum may be some additional injury or violence. (3) The chief cause of involvement of the spinal cord is the formation of a large amount of tuberculous granulation tissue in the canal, the so-called tubercular granuloma; 98 per cent. of the cases of paraplegia are due to this encroachment by granulation tissue. In some cases the term granuloma is particularly appropriate, for the amount of connective tissue is sometimes extremely large, so that the tumour mass is then very hard, and may be removable only with a knife or a sharp spoon.

A further cause of paraplegia is pachymeningitis, and I take it that a greater or less degree of this is present whenever there is granulation tissue in the canal, with thickening of the dura. This pachymeningitis is localised, not spreading far above or below the seat of disease. In the majority of cases the inner surface of the dura mater escapes, the outer being alone affected: that is, the dura affords a satisfactory protection to the cord itself. Cases have been described of tuberculous myelitis and leptomeningitis as the result of tuberculous caries.

The question as to whether the affection of the cord in paraplegia cases is a tuberculous myelitis or not has led to a great deal of discussion; but I do not propose to deal with it beyond saying that myelitis in children is more likely to be due to Pott's disease than to any other cause. There are three theories in regard to what takes place in the cord, all of which are to some extent true: (1) The first supposes that the process is an ischæmia of the cord due to pressure on the blood vessels, with subsequent necrosis. (2) The next ascribes the symptoms to pressure on the lymph spaces, giving rise to stasis and serous effusion with œdema followed by secondary degeneration. (3) The third regards the effects as those of simple mechanical pressure on the nerve elements, which causes a primary degeneration.

No matter which of these takes place, the end result is the same in every case—namely, destruction of the nerve elements, and secondary degeneration which gradually increases unless an arrest of the disease is brought about. In addition to involvement of the cord the nerve roots are also affected where they pass through the caseous mass.

### Symptoms.

Now let me say a few words about symptoms. The onset of the symptoms which point to spinal cord involvement is as a rule insidious; but it may be acute, as when abscesses or bony sequestra give rise to rapid paraplegia. I want to repeat that such a paralysis may be the first symptom of the disease. Having come on in one or other of these ways, the stages of the disease are similar to those of compression paraplegia from any other cause. We are in the habit of describing three stages: (1) Neuralgic pains more or less severe, "girdle sensation," and slight loss of power are present. The girdle sensation and shooting pains are due to pressure upon the nerve roots. In passing let me remark that in children who are the subjects of unrecognised Pott's disease the complaint of stomach-ache is a point in the diagnosis which is often not fully appreciated. In this early stage loss of power is usually shown by complaints of being easily tired, or, in children, by disability for playing or running about. The tendency to hitch the toes or drag the feet in walking is often very marked. (2) In the second stage there is paralysis of motion and of sensation. The paralysis is a gradually increasing one, provided means are not taken to stay the progress of the disease. Motor power is much earlier affected than sensation. The explanation of this is simply an anatomical one, for the anterior and lateral columns of the cord are much closer than the posterior columns to the commonest position of the carious foci; in the rare cases of posterior spinal tuberculosis sensation is affected before motion. The sphincters also become weak: the onset of this trouble is variable, for it is sometimes early, sometimes late, or absent. (3) In the last stage of all there is system degeneration up and down in the spinal cord, and trophic disorders appear, such as bedsores and the like.

### Treatment.

There is one form of treatment which should be in everyone's mind, and that is prophylaxis. The occurrence of paraplegia is just as much a reproach as is that of angular deformity. What are the chief points in prophylaxis which we should study to prevent the onset of paralysis? The first is *early diagnosis*, before angular curvature has occurred; in the very first stages, when the only indications may perhaps be pain and muscular spasm. The other is *prolonged rest in the recumbent position*. The cases should not be put off with plaster jackets or spinal braces: they should have prolonged rest treatment until one is satisfied that the disease is entirely cured. Then—and only then—should some mechanical appliance be ordered and the patient allowed up. If this were done in all cases very few would come to a paraplegic condition.

Having a case of paraplegia to deal with, how are we going to treat it? The general treatment is, of course, the same as for tuberculous disease anywhere else. The details of outdoor and of medicinal treatment I need not go into. How long should we go on with mechanical before adopting operative treatment? All early cases of paraplegia, when mechanical treatment can be *intelligently* carried out, should be given a fair trial of it. It should be continued just as long as there are any signs of improvement in the condition of the cord. In many cases this will require months or years of treatment, which renders it difficult or impossible in hospitals owing to the limited bed-space.

### Operations.

What are the indications and contra-indications for the operative treatment? Many surgeons of repute claim with truth that all these cases should be treated as tuberculous abscesses of bone are treated in other parts of the body—that is, by operation. But there are one or two difficulties in the way. Firstly, many cases get well without operation. Secondly, there are anatomical obstacles to getting away the whole of the diseased tissues, especially in the usual anterior cases in the cervical and dorsal regions.

There are, however, certain cases in which operation is distinctly indicated. They are those of acute onset to which I have already referred: all cases of abscess formation or of pressure by a sequestrum should be submitted without delay to operation. In passing I should like to refer to the cases in which acute symptoms do not necessarily indicate operation: that is, in which the acute symptoms are due to changes in the cord itself, and not to aggravation of the local tuberculous disease. In trying to decide the question of operation one has to consider several points. Of primary importance is the actual condition of the cord itself. Another point is the prospect of recovery of the functions of the cord under mechanical treatment, and a third is the prospect of recovery of the functions of the cord if the pressure is removed by operation. The degree of pressure is also of importance, and the length of time during which it has been exerted; so also is the liability of the cord to degeneration. These are all varying factors whose effect it is very difficult to estimate.

Of the general contra-indications I would say that one is tuberculous disease in other parts of the body. Another includes the cases in which mechanical treatment has not been adequately and intelligently carried out. Lack of means to perform an operation aseptically and to nurse the case properly afterwards are also to be mentioned.

Of indications for operation the chief are acute onset, and thorough trial of mechanical treatment without improvement. Posterior cases where a focus can be entirely removed are also especially suited for operation. It is hardly fair to a surgeon to ask him to operate with any prospect of doing good when the patient has been allowed to linger on until the functions of the cord are so affected that they can never be recovered, and complications such as cystitis and bedsores have supervened. When sensation is not affected, but motor power only, better results are obtained than when the reverse is the case.

(Several cases were shown at the end of the lecture.)

